# Sparse multi‐trait genomic prediction under balanced incomplete block design

**DOI:** 10.1002/tpg2.20305

**Published:** 2023-02-22

**Authors:** Osval A. Montesinos‐López, Brandon A. Mosqueda‐González, Josafat Salinas‐Ruiz, Abelardo Montesinos‐López, José Crossa

**Affiliations:** ^1^ Facultad de Telemática Universidad de Colima Colima Mexico; ^2^ Centro de Investigación en Computación (CIC) Instituto Politécnico Nacional (IPN) Mexico City Mexico; ^3^ Colegio de Postgraduados Campus Córdoba Amatlán de los Reyes Mexico; ^4^ Centro Universitario de Ciencias Exactas e Ingenierías (CUCEI) Universidad de Guadalajara Guadalajara Mexico; ^5^ International Maize and Wheat Improvement Center (CIMMYT) Edo. de México Mexico; ^6^ Colegio de Postgraduados Montecillos Mexico

## Abstract

Sparse testing is essential to increase the efficiency of the genomic selection methodology, as the same efficiency (in this case prediction power) can be obtained while using less genotypes evaluated in the fields. For this reason, it is important to evaluate the existing methods for performing the allocation of lines to environments. With this goal, four methods (M1–M4) to allocate lines to environments were evaluated under the context of a multi‐trait genomic prediction problem: M1 denotes the allocation of a fraction (subset) of lines in all locations, M2 denotes the allocation of a fraction of lines with some shared lines in locations but not arranged based on the balanced incomplete block design (BIBD) principle, M3 denotes the random allocation of a subset of lines to locations, and M4 denotes the allocation of a subset of lines to locations using the BIBD principle. The evaluation was done using seven real multi‐environment data sets common in plant breeding programs. We found that the best method was M4 and the worst was M1, while no important differences were found between M3 and M4. We concluded that M4 and M3 are efficient in the context of sparse testing for multi‐trait prediction.

## Core Ideas

- Genomic selection increases genetic gain in plant breeding programs.
- Sparse testing is essential to increase genomic prediction accuracy.
- Incomplete block design for sparse testing improves genomic‐enables prediction accuracy.

**Abbreviations**
APC: average Pearson's correlation
Bed2IR: bed planting with two irrigations
Bed5IR: bed planting with five irrigations
BIBD: balanced incomplete block design
DTHD: number of days from germination to 50% spike emergence
DTMT: days to maturity
EHT: early heat
Flat5IR: flat planting and five irrigations
FlatDrip: flat planting with drip irrigation
GE: genotype × environment
GY: grain yield
LHT: late heat
MAF: minor allele frequency
METs: multi‐environmental trials
NPP: pods per plant
NRMSE: normalized root mean squared error
PYPP: pod yield per plant
SNP: single‐nucleotide polymorphism
SYPP: seed yield per plant
TASSEL: Trait Analysis by Association Evolution and Linkage
YPH: yield per hectare

## 1 INTRODUCTION

Genomic selection (GS) (Meuwissen et al., 2001) increases genetic gain in plant breeding programs. Therefore, GS is being implemented in many crops such as wheat (*Triticum* L.), maize (*Zea mays* L.), cassava (*Manihot* Mill.), rice (*Oryza sativa* L.), chickpea (*Cicer arietinum* L.), and groundnut (*Apios* Fabr.), among others (Crossa et al., 2017; Huang et al., 2019; Roorkiwal et al., 2016; Wolfe et al., 2017). Nevertheless, the real, practical implementation of GS is still complex because many genetic and environmental factors affect the prediction accuracy in the GS methodology (Crossa et al., 2017; Cuevas et al., 2017).

For the reason above, many approaches have been evaluated to increase the efficiency of the GS methodology, some of which are enumerated hereafter: Approach 1 is centered on evaluating many statistical machine learning methods, which have been used in GS to identify robust methods that guarantee high accuracy. For this reason, mixed models, penalized methods like Ridge regression and Lasso Regression, Bayesian methods (BayesA, BayesB, BayesC, Bayesian Lasso, GBLUP, etc.), random forest, Gradient boosting machine, artificial deep neural networks, and others have been tested in GS (Montesinos‐López, Montesinos‐López, & Crossa, 2022). Approach 2 consists of guaranteeing high‐quality phenotypic and genotypic data to obtain high‐quality outputs. Approach 3 focuses on optimizing the training and testing set to guarantee predictions in the testing set according to the complexity of the traits; many publications corroborate that the composition of the training sets is essential in improving prediction accuracy (Crossa et al., 2017; Rio et al., 2022). Under this third approach, many strategies for selecting a training set given a testing set have been proposed, while many other strategies for creating a testing set given a training set have also been studied and evaluated (Lopez‐Cruz et al., 2020; Lopez‐Cruz & de los Campos, 2021)

Under the third approach, when the genotypes are evaluated in multi‐environmental trials (METs), candidate genotypes are evaluated under different environmental conditions, and breeders can select stable genotypes across environments and in specific environments since the genotype × environment (GE) interaction is modeled. Nevertheless, evaluating all genotypes in all the environments is financially costly as it requires extensive field‐testing evaluations (Smith, Butler, et al., 2015; Smith, Ganesalingam, et al., 2015).

Consequently, to reduce plant breeding costs in the implementation of the GS methodology in METs, “sparse testing” strategies have been proposed. Sparse testing means that some genotypes have been evaluated in some environments but not in others. As such, the use of sparse testing has the potential to increase the number of environments under evaluation in early‐stage yield testing without a significant increase in the cost of phenotyping. Since sparse testing does not evaluate all lines in each environment and only a fraction of lines are allocated for each environment, it is very attractive for improving the following two goals: (a) increasing the number of lines tested across multiple, diverse environments and (b) increasing the number of testing environments while maintaining the same selection intensity (Atanda et al., 2021; Burgueño et al., 2012; Jarquin et al., 2020).

For a sparse testing, different approaches could be followed for distributing lines in environments. Let us suppose 10 lines and four locations with half of lines (5) for training and half (5) for testing training. The most simple sparse testing method, M1, consists of allocating a subset of genotypes to all locations (e.g., the same five lines tested in four locations); M2 allocates a subset of lines in such a way that some lines are shared between some locations (e.g., four locations with the same three lines, three locations with two different lines and one line tested in only one location); M3 randomly allocates lines to locations such that all five lines are randomly distributed in each location (e.g., some lines are in three locations, other lines are in two locations and others in one location); and M4 allocates a subset of lines to locations followed a balanced incomplete block design (BIBD) scheme. Thus, the difference between M2 and M4 is the arrangement of the subset of lines (BIBD, M4 vs. random M2).

The BIBD method for sparse allocations of lines to locations is very attractive since it uses all the virtues of experimental designs, which are powerful tools that have been long used in plant breeding programs to improve the accuracy of parameter estimates. The BIBD (M4) proposed by Montesinos‐López, Montesinos‐Lopez, Acosta, et al. (2022) showed through five real data sets that the prediction accuracy of the sparse testing method is better than the random allocation of lines to locations (M3); however, this method was proposed and evaluated under a univariate (single‐trait) framework and was only compared to M3. It should be noted that these four sparse testing methods (M1, M2, M3, and M4) have not yet been compared in the same study.

**Core Ideas**

- Genomic selection increases genetic gain in plant breeding programs.
- Sparse testing is essential to increase genomic prediction accuracy.
- Incomplete block design for sparse testing improves genomic‐enables prediction accuracy.

Therefore, based on the previous considerations, the main objective of this study is to evaluate the four methods of sparse testing under a multivariate (multi‐trait) context, that is, when we are interested in predicting more than one response variable simultaneously. For comparing the genomic prediction ability of the four methods of sparse testing (including the BIBD, M4), we study seven real multiple‐traits and multi‐environments data sets.

## 2 MATERIALS AND METHODS

### 2.1 Data sets

In this section, we present the seven data sets used for benchmarking the four sparse allocation methods that are described in the next section. The quality control for the genomic data consists of removing markers that had more than 15% missing values and those with a minor allele frequency (MAF) of < 0.05.

#### 2.1.1 Data set 1. Disease

In this data set with 438 wheat lines, three traits (diseases) were measured: *Pyrenophora tritici‐repentis*, which causes a disease known as yellow spot, also known as yellow leaf spot, tan spot, yellow leaf blotch, or helminthosporiosis; *Parastagonospora nodorum*, a major wheat fungal pathogen that affects the leaves and other parts of the plants; and *Bipolaris sorokiniana*, the cause of seedling diseases, common root rot and spot blotch in several crops such as barley and wheat. Over a long period during the same year, these 438 lines were evaluated in the greenhouse for several replicates, which were subsequently considered environments (Env1, Env2, Env3, Env4, Env5, and Env6) as the experiments were established in different greenhouses and planting dates. For the three traits measured, the total number of observations was 438×6=2628. It should be noted that this data set was also used by Montesinos‐Lopez et al. (2020). The response was measured on a continuous scale from 1 (resistance) to 5 (susceptible).

DNA samples were genotyped using 67,436 single nucleotide polymorphisms (SNPs). For a given marker, the genotype for each line was coded as the number of copies of a designated marker‐specific allele carried by the line (absence = zero and presence = one). The markers that had more than 15% missing values were removed, as well as markers with MAF < 0.05. A total of 11,617 SNPs were still available for analysis after quality control and imputation.

#### 2.1.2 Data set 2 (EYT_1), data set 3 (EYT_2), data set 4 (EYT_3), and data set 5 (EYT_4)

These four data sets were collected by the Global Wheat Program of the International Maize and Wheat Improvement Center (CIMMYT) and belong to elite yield trials (EYT) established in four different cropping seasons with four environments. Data set 2 (EYT_1) is from cycle 2013–2014, data set 3 (EYT_2) is from cycle 2014–2015, data set 4 (EYT_3) is from cycle 2015–2016, and data set 5 (EYT_4) is from cycle 2016–2017. The EYT data sets 1, 2, 3 and 4 contain 766, 775, 964 and 980 lines, respectively.

The experimental design used was an alpha‐lattice design, and the lines were sown in 39 trials, each covering 28 lines and two checks in six blocks with three replications. Several traits were available for some environments and lines in each data set. In this study, we included four traits measured for each line in each environment: days to heading (DTHD, number of days from germination to 50% spike emergence), days to maturity (DTMT, number of days from germination to 50% physiological maturity or the loss of the green color in 50% of the spikes), PH, and GY. For full details on the experimental design and how the best linear unbiased estimated were computed, see Juliana et al. (2018). Since wheat lines included in data sets 2–5 are different (as they included different years), we decided to treat each cycle (year) separately.

The lines examined in data sets were evaluated in four environments. For data set 2 (EYT_1), the environments were bed planting with five irrigations (Bed5IR), early heat (EHT), flat planting and five irrigations (Flat5IR), and late heat (LHT). For data set 3 (EYT_2), the environments were bed planting with two irrigations (Bed2IR), Bed5IR, EHT, and Flat5IR. For data set 4 (EYT_3), the environments evaluated were Bed2IR, Bed5IR, Flat5IR, and flat planting with drip irrigation (FlatDrip). For YET_4 data set 5, the four environments were Bed5IR, EHT, Flat5IR, and FlatDrip. Genome‐wide markers for the 3495 (776 + 775 + 964 + 980) lines in the four data sets were obtained through genotyping‐by‐sequencing (Elshire et al., 2011) at Kansas State University using an Illumina HiSeq2500. After filtering, 2038 markers were obtained from an initial set of 34,900 markers. The imputation of missing markers data was carried out using LinkImpute (Money et al., 2015) and implemented in TASSEL (Trait Analysis by Association Evolution and Linkage) version 5 (Bradbury et al., 2007). Lines that had more than 50% missing data were removed, and 3495 lines were used in this study (776 lines in the second data set, 775 lines in the third data set, 964 lines in the fourth data set, and 980 lines in the fifth data set).

#### 2.1.3 Data set 6. Groundnut

The phenotypic data set reported by Pandey et al. (2020) includes information on the phenotypic performance of 318 groundnut lines for various traits in four locations. We assessed genomic‐enabled predictions for the following four traits: pods per plant (NPP), pod yield per plant (PYPP) measured in grams, seed yield per plant (SYPP) measured in grams, and yield per hectare (YPH) measured in kilograms. The locations are denoted as Aliyarnagar_R15, Jalgoan_R15, ICRISAT_R2015, and ICRISAT_PR15‐16. The data set is balanced, giving a total of 1272 assessments with each line included once in each location. Marker data were available for all lines, and 8268 single‐nucleotide polymorphism (SNP) markers remained after quality control (with each marker coded with 0, 1, or 2).

#### 2.1.4 Data set 7. Japonica

Monteverde et al. (2019) also reported a rice data set, belonging to the tropical Japonica population with four traits (GY; percentage of head rice recovery; percentage of chalky grain; PH, plant height) as for the Indica population (data set 7) but containing 4 years (2010, 2011, 2012, and 2013). A total of 127 lines were evaluated each year, and 16,383 SNP markers remained after coding the marker as 0, 1, and 2. In this vein, a total of 508 assessments were evaluated in the four environments. In this data set, the lines evaluated were 127.

### 2.2 Sparse allocation methods of lines to environments

In this section, we will describe the four sparse testing methods used to allocate lines to locations.

#### 2.2.1 Method 1 (M1): Allocation of a fraction (subset) of lines in all locations

**FIGURE 1:**
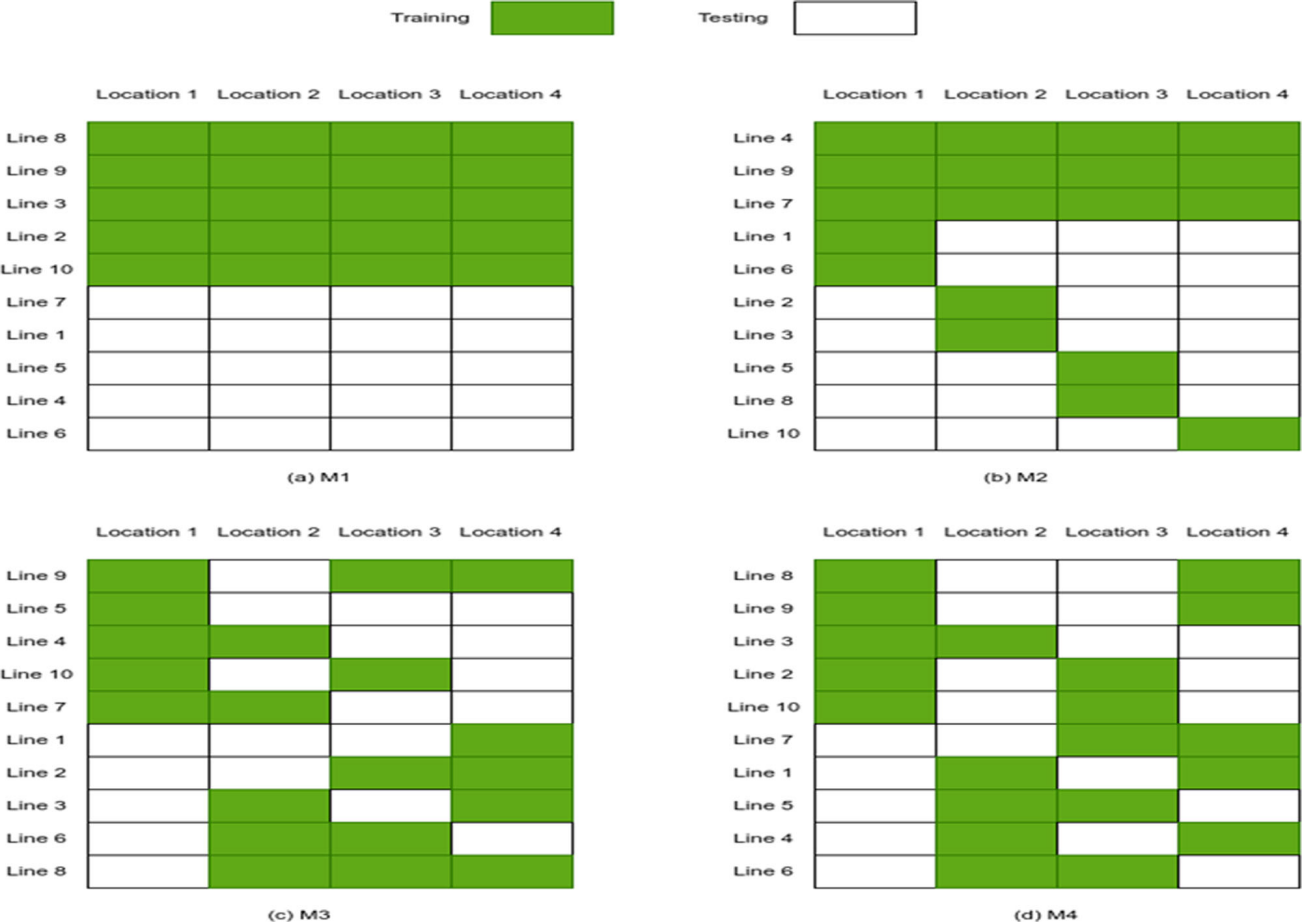
Allocation methods with 10 lines and four locations for a partition with 50% of lines as training and 50% of lines as testing. M1 denotes the allocation of some lines in all locations, M2 denotes the allocation of a fraction (subset) of lines with some shared lines in locations, M3 denotes the random allocation of some lines to locations, and M4 denotes the allocation of a fraction of lines to locations using the balanced incomplete block design method

This is the simplest allocation method where a fraction (subset) of lines is taken, then allocated in all locations as a training set, and the remaining lines are used as the testing set. In Figure 1a, we can see how the partition is formed with this method. The green color represents the lines used as the training set, and the white color represents the lines used as the testing set. Training lines are grouped at the beginning of Figure 1a, but lines are not in numerical order, thus showing that they were randomly selected.

#### 2.2.2 Method 2 (M2): Allocation of a fraction (subset) of lines with some shared lines in locations

Similar to M1, M2 takes a fraction (subset) of lines to be used as training set and the remaining as testing set in one location. For the other locations, the testing lines are divided into the number of locations—one part that is ideally with the same size and one part is interchanged from testing to training for each location. In this way, each location shares most of the training lines but contains some lines that are only in the training, as show in Figure 1b.

#### 2.2.3 Method 3 (M3): Random allocation of a fraction of lines to locations

Starting from a balanced data set with *J* lines and *I* locations, the conformation of the random allocation of lines to locations was done in such a way that approximately each line will be repeated in *r* out of *I* locations, and all locations will be of the same size (*k*). The algorithm (Montesinos‐López, Montesinos‐Lopez, Acosta, et al., 2022) of this random allocation is as follows:

1. First, we compute $k\;=\frac{J_{\times r}}{I}\;$ (least integer greater than or equal to $\frac{J_{\times r}}{I}$). Then, *k* lines out of *J* lines are randomly allocated to the first location, and *r* is the number of replications of each line.
2. Next, for the second location, *k* out of the *J* lines were once again randomly allocated.
3. This process is repeated until the *I*th location is completed, with the caveat that the lines allocated to a particular location are only present in less than or equal to *r* locations, ideally in exactly *r* locations. The lines that do not satisfy this restriction are not candidates for being allocated to a particular location.

In Figure 1c, one example of this method is shown when used with 10 lines and four locations. As can be seen, some locations appear up to three times, while others appear only twice or once since it is a random process.

### 2.3 Method 4 (M4): Allocation of lines to locations using the BIBD

A balanced BIBD design is where all pairs of lines occur together within a location and an equal number of times (λ). In general, we will specify $\lambda_{jj}$ as the number of times line *j* occurs within a location. To generate this sparse allocation of lines to locations (Montesinos‐López, Montesinos‐Lopez, Acosta, et al., 2022), we can use the function find.BIB() using the R package crossdes (R Core Team, 2022). Let us suppose there are J=10 lines and I=4 locations, this means that we need 40 plots to allocate the 10 lines to the four locations under a complete block design. However, we will use a BIBD and a training set of size N_TRN=20, which accounts for (50%) of the total plots required under a randomize complete block design. Therefore, the number of lines by locations can be obtained by solving (kI=N_TRN) for *k*, which results in k=N_TRN/I. This means that k=20/4=5 lines per location. Then, the corresponding elements for the training set can be obtained with the function find.BIB(10, 4, 5) using the package crossdes. The numbers used in the function find.BIB() denote the lines, the locations, and the lines per locations, respectively.

Figure 1d shows how a particular allocation may look using this method. One important aspect is that all lines are used at the same time (2) and all locations contain the same number of lines (5). This is an important difference with respect to method M3 where some lines are tested in three locations, other lines are tested in two locations and one line is evaluated in only one location.

### 2.4 Statistical model

This model is given by

(1) $$Y=1_{n}\mu^{T}+X_{E}\beta_{E}+Z_{L}g+Z_{EL}\mathrm{gE}+\varepsilon ,$$

where ***Y*** is the matrix of phenotypic response variables of order $n\times n_{T}$ and ordered first by environments and then by lines; $n_{T}$ denotes the number of traits; 1_*n*_ is a matrix of ones of order $n\times n_{T}$, where $n_{T}$ denotes the number of traits; $\mu^{T}$ is a vector of intercepts for each trait of length $n_{T}$, *T* denotes the transpose of a vector or matrix, that is, $\mu ={\left[\mu_{1},\text{…},\mu_{n_{T}}\right]}^{T};\;X_{E}$ is the design matrix of environments of order n×I; *I* denotes the number of environments (locations); $\beta_{E}$ is the matrix of beta coefficients for environments with a dimension of $I\times n_{T}$; ***Z***_*L*_ is the design matrix of lines of order n×J; *J* denotes the number of lines; ***g*** is the matrix of random effects of lines of order $J\times n_{T}$ distributed as $g∼MN_{J\times n_{T}}\left(0,G,\Sigma_{T}\right)$, that is, with a matrix‐variate normal distribution with parameters M=0, U=G and $V\;=\Sigma_{T}\;$; ***G*** is the genomic relationship matrix as proposed by VanRaden (2008) built with marker data of order J×J; $\Sigma_{T}$ is the variance–covariance matrix of traits of order $n_{T}\times n_{T}$; $Z_{EL}$ is the design matrix of the GE interaction of order n×JI; ***gE*** is the matrix of the GE interaction random effects distributed as $\mathrm{gE}∼MN_{JI\times n_{T}}\left(0,G⊗\Sigma_{E},\Sigma_{T}\right)$, where $\Sigma_{E}$ is a diagonal variance–covariance matrix of environments of orderI×I and $G⊗\Sigma_{E}$ is the Kronecker product of the *l*th type of kernel matrix of lines and the environmental relationship matrix; and ε is the residual matrix of dimension $n\times n_{T}$ distributed as $\varepsilon ∼MN_{n\times n_{T}}\left(0,I_{IJ},R\right)$, where ***R*** is the residual variance–covariance matrix of order $n_{T}\times n_{T}$. The implementation of this model was carried out in the BGLR library of Pérez and Campos (2014).

Under the four types of allocation methods, we use the notation *J* as the number of lines, *k* as the number of lines per location, *I* as the number of locations, and *r* as the number of replications of each line *j* in the entire design. It should be noted that in BIBDs *k* will be less than *J*, since not all the lines in each location can be assigned. An equal number of entry replication is the best way to ensure minimum variance when making all possible pairwise comparisons. Therefore, since $r_{i}=\;r$ for all lines, the total number of observations in the experiment is *N*, where N=J×r=I×k.

### 2.5 Cross‐validation strategy

To evaluate and compare the predictive performance of the allocation methods, we used cross‐validation with 10 partitions and 50% of the data for training and 50% for testing. The average normalized root mean squared error (NRMSE) was computed (Montesinos‐López, Montesinos‐López, & Crossa, et al., 2022) with the 10 random partitions, and this metric was used to assess the predictive performance in each data set for each allocation method. NRMSE is defined as:

$$\mathrm{NRMSE}\left(y,\hat{y}\right)=\frac{\sqrt{\frac{1}{n}\sum_{i=1}^{n}{\left(y_{i}-\hat{y_{i}}\right)}^{2}}}{\bar{y}},$$

where $y_{i}$ is the *i*th observed value, $\hat{y_{i}}$is the *i*th predicted value, and *n* is the total number of observations. Additionally, the average Pearson's correlation (APC) was reported as a metric of prediction accuracy using observed and predicted values. Across locations, the NRMSE and Pearson's correlations in each partition was computed between averages of true and predicted phenotypic values over locations; subsequently, the average of the NRMSEs and APC of the 10 partitions was reported as prediction performance in each data set. Since the allocation methods were evaluated with a multi‐trait model, the NRMSE was computed for each trait separately and then averaged within each fold.

## 3 RESULTS

The results are given in seven sections, one for each data set, and in each section we compare the prediction performance obtained with the four methods of sparse allocations of lines to locations. This comparison was made in terms of the NRMSE and the APC.

### 3.1 Data set 1 (disease)

**FIGURE 2:**
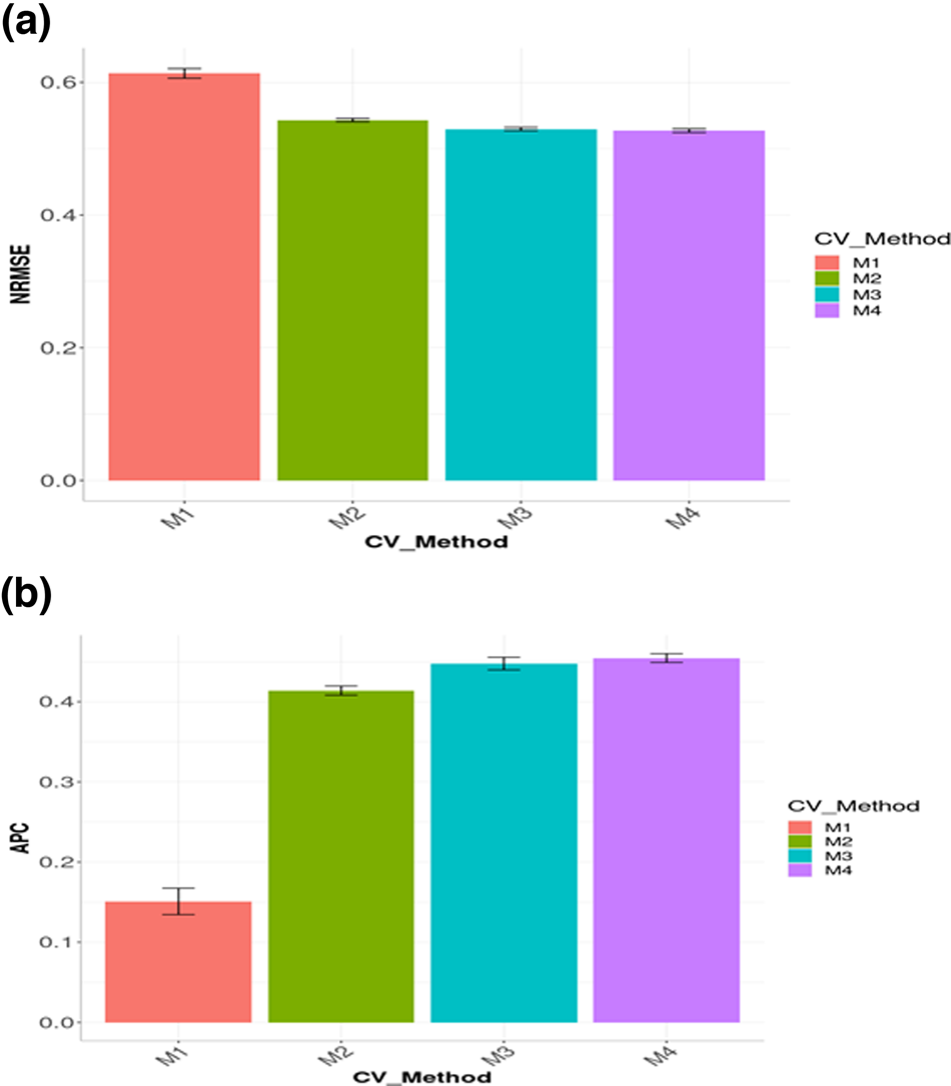
Prediction performance in terms of normalized root mean squared error (NRMSE) (A) and average Pearson's correlation (APC) (B) for data set 1 (disease). M1 denotes the allocation of lines in all locations, M2 denotes the allocation with some shared lines in locations, M3 denotes the random allocation of lines to locations, and M4 denotes the allocation of lines to locations using the balanced incomplete block design (BIBD) method. Ten partitions were implemented with 50% of the data for training and 50% for testing

Under this data set, the best results were obtained with method M4 (BIBD method) in terms of NRMSE, according to results of Table A1 and Figure 2A. This best method outperformed M1, M2, and M3 by 14.05%, 2.92% and 0.45%, respectively. In terms of APC, this gain is also observed with the same pattern: M4 gives the greatest APC (0.4545), while M1 gives the lowest (0.1509) (Figure 2B).

### 3.2 Data set 2. EYT_1

**FIGURE 3:**
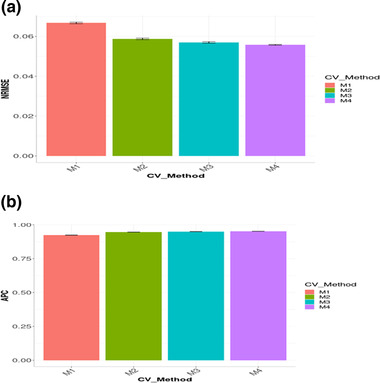
Prediction performance in terms of normalized root mean squared error (NRMSE) (A) and average Pearson's correlation (APC) (B) for data set 2 (EYT_4). M1 denotes the allocation of lines in all locations, M2 denotes the allocation with some shared lines in locations, M3 denotes the random allocation of lines to locations, and M4 denotes the allocation of lines to locations using the balanced incomplete block design (BIBD) method. Ten partitions were implemented with 50% of the data for training and 50% for testing

Like the results in data set 1, the best prediction performance was obtained with M4, followed by M3, M2, and finally M1. M4 was superior to M1, M2, and M3 by 16.46%, 5.1%, and 2.1%, respectively (Figure 3A). For APC, the same pattern is observed since M4 produces the highest APC followed by methods M3, M2, and M1, respectively (Figure 3B). Details are given in Table A1.

### 3.3 Data set 3. EYT_2

**FIGURE 4:**
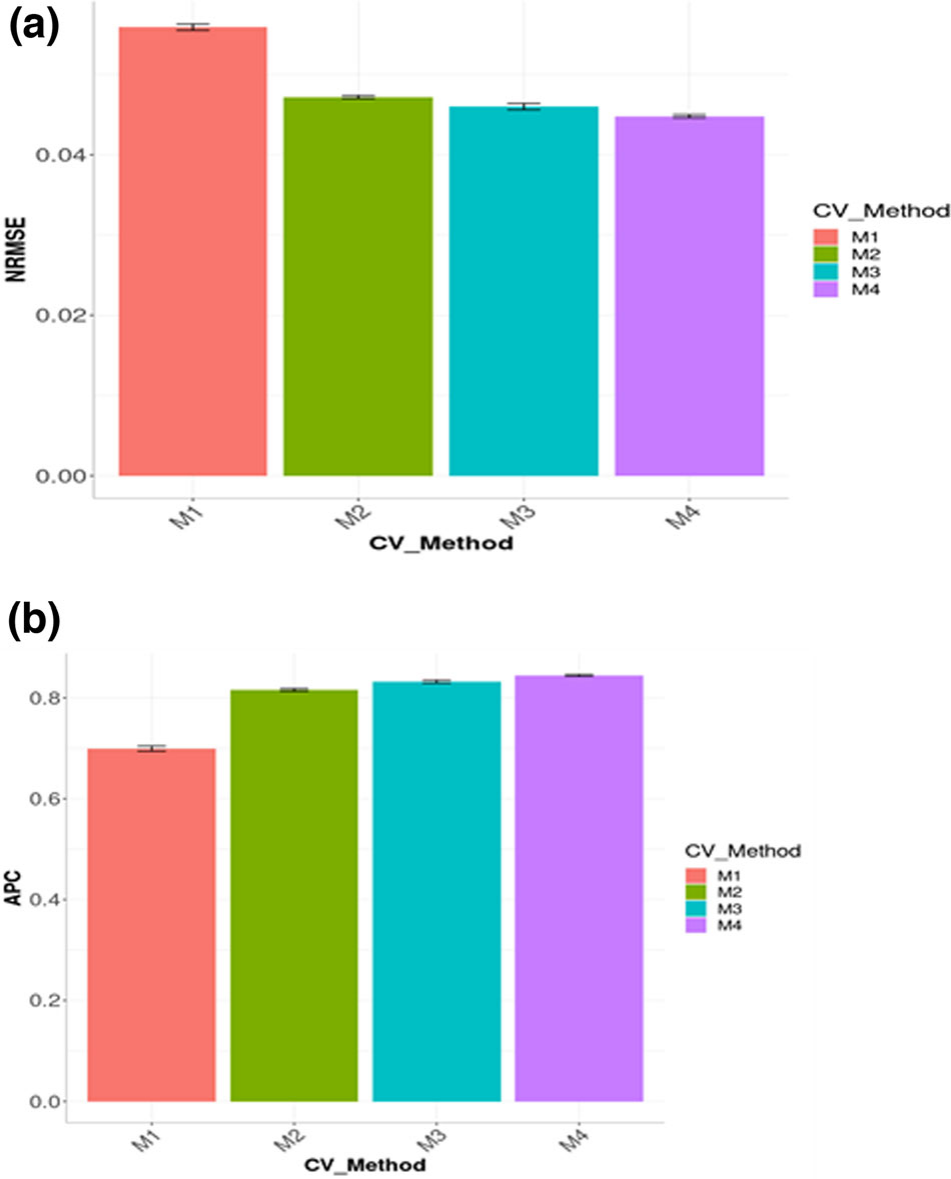
Prediction performance in terms of normalized root mean squared error (NRMSE) (A) and average Pearson's correlation (APC) (B) for data set 3 (EYT_1). M1 denotes the allocation of lines in all locations, M2 denotes the allocation with some shared lines in locations, M3 denotes the random allocation of lines to locations, and M4 denotes the allocation of lines to locations using the balanced incomplete block design (BIBD) method. Ten partitions were implemented with 50% of the data for training and 50% for testing

This data set contains four traits (DTHD, DTMT, GY, and Height), and the results show that M4 (BIBD) gives the lowest error in terms of NRMSE across traits. Comparing the M4 method with all other methods, M4 is more efficient than M1 by 19.85%, M2 by 5.08%, and M3 by 2.6% (Figure 4A and Table A1). As expected, the methods with lowest errors are the ones with higher APC, with M4 having an APC = 0.8444, M3 with an APC = 0.8322, M2 with an APC = 0.8156, and finally M1 with an APC = 0.6994 (Figure 4B).

### 3.4 Data set 4. EYT_3

**FIGURE 5:**
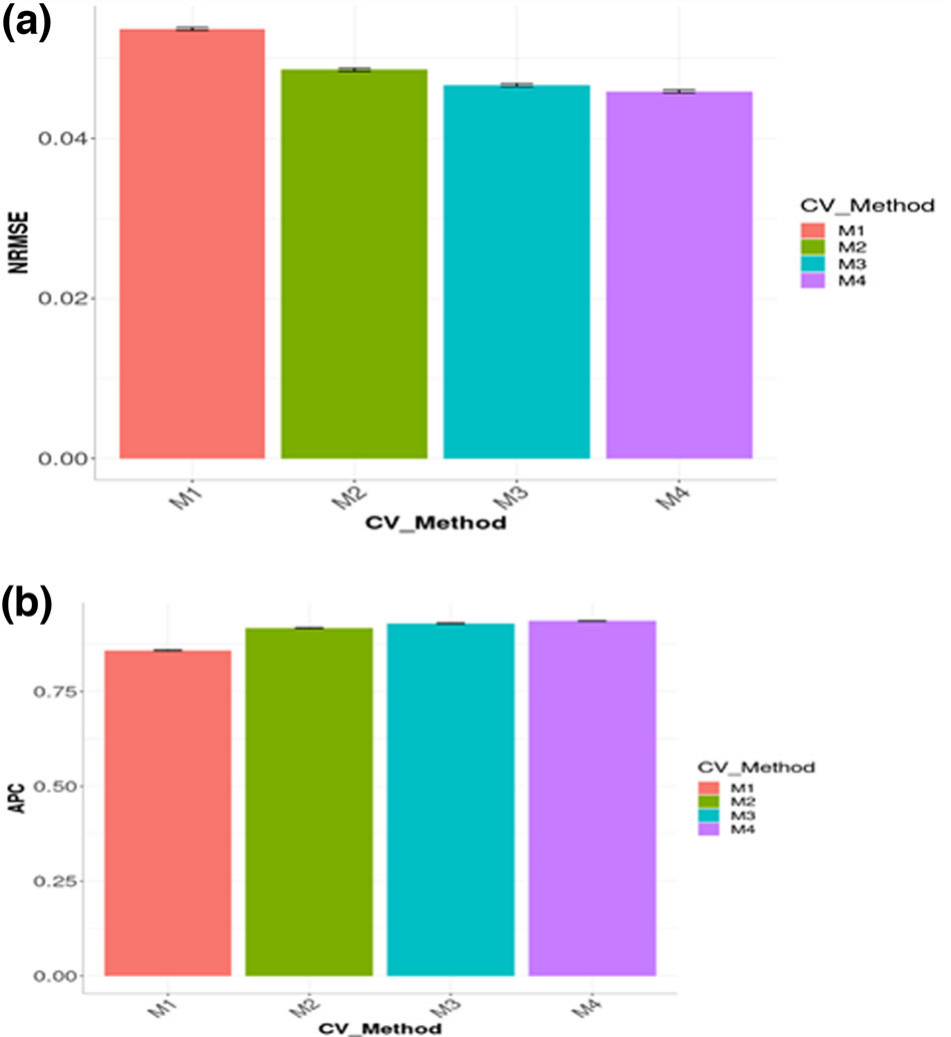
Prediction performance in terms of normalized root mean squared error (NRMSE) (A) and average Pearson's correlation (APC) (B) for data set 4 (EYT_2). M1 denotes the allocation of lines in all locations, M2 denotes the allocation with some shared lines in locations, M3 denotes the random allocation of lines to locations, and M4 denotes the allocation of lines to locations using the balanced incomplete block design (BIBD) method. Ten partitions were implemented with 50% of the data for training and 50% for testing

In Figure 5A, we present the results for data set 4 in terms of NRMSE where we can observe that M4 and M3 were superior to M1 and M2 since they present lower prediction errors. Specifically, M4 is superior to M3 by 1.71%, to M2 by 5.55%, and to M1 by 14.52%, according to Figure 5A and Table A1. In terms of APC, the results in Figure 2 show that APC is higher for those methods with lower NRMSE. For example, M4 gives the lowest NRMSE (0.0459) and the highest APC (0.9363); on the other hand, M1 gives the highest NRMSE (0.0537) and the lowest APC (0.8587) (Figure 5). See details in Table A1.

### 3.5 Data set 5. EYT_4

**FIGURE 6:**
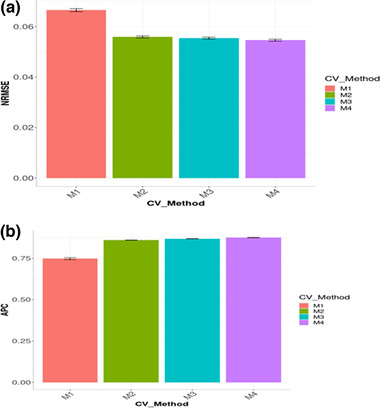
Prediction performance in terms of normalized root mean squared error (NRMSE) (A) and average Pearson's correlation (APC) (B) for data set 5 (EYT_7). M1 denotes the allocation of lines in all locations, M2 denotes the allocation with some shared lines in locations, M3 denotes the random allocation of lines to locations, and M4 denotes the allocation of lines to locations using the BIBD method. Ten partitions were implemented with 50% of the data for training and 50% for testing

This is the largest data set of all those evaluated. In this data set, the gain of methods M2, M3, and M4 with respect to M1 is considerably large (Figure 6A). M2, M3, and M4 give similar results to each other, but the gain in terms of NRMSE to M1 is of 15.94% (M2), 16.69% (M3), and 17.89% (M4), respectively, where we can observe that the largest gain was observed with the method M4 yet again. In terms of APC, the gain of M2, M3, and M4 is also displayed in Figure 6B, where we can observe that these three methods have similar APC, but M1 is considerably lower. Details are given in Table A1.

### 3.6 Data set 6. Groundnut

**FIGURE 7:**
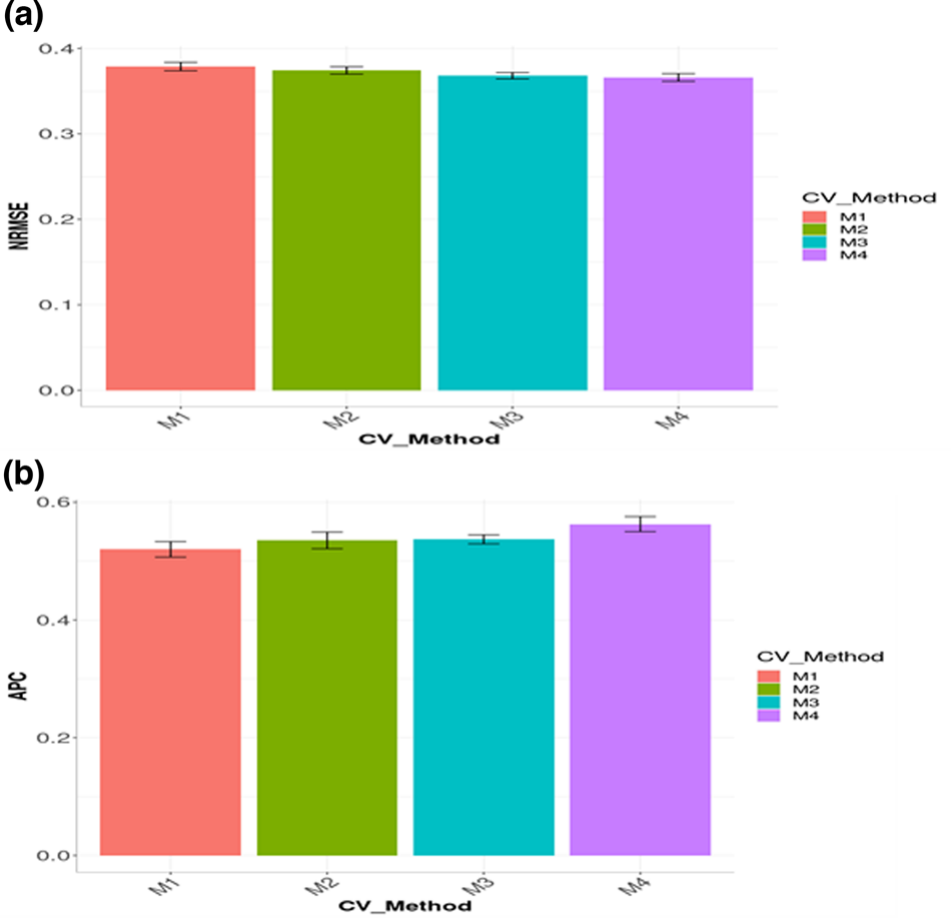
Prediction performance in terms of normalized root mean squared error (NRMSE) (A) and average Pearson's correlation (APC) (B) for data set 6 (Groundnut). M1 denotes the allocation of lines in all locations, M2 denotes the allocation with some shared lines in locations, M3 denotes the random allocation of lines to locations, and M4 denotes the allocation of lines to locations using the balanced incomplete block design (BIBD) method. Ten partitions were implemented with 50% of the data for training and 50% for testing

The results for groundnut data set follow the same patterns described in the previous data sets but with smaller differences. As in other data sets, M4 gives the best NRMSE (0.366) that is superior to M1 by 3.37%, M2 by 2.19%, and M3 by 0.57% (Figure 7A). As can be seen in Figure 7A, the NRMSEs are similar but M4 is still the best. In terms of APC, the gain of M4 with regard to M1 was of 4.92%; with respect to M2, the gain was of 4.6%, and compared with M3 the gain was of 2.12% (Figure 7B). See details in Table A1.

### 3.7 Data set 7. Japonica

**FIGURE 8:**
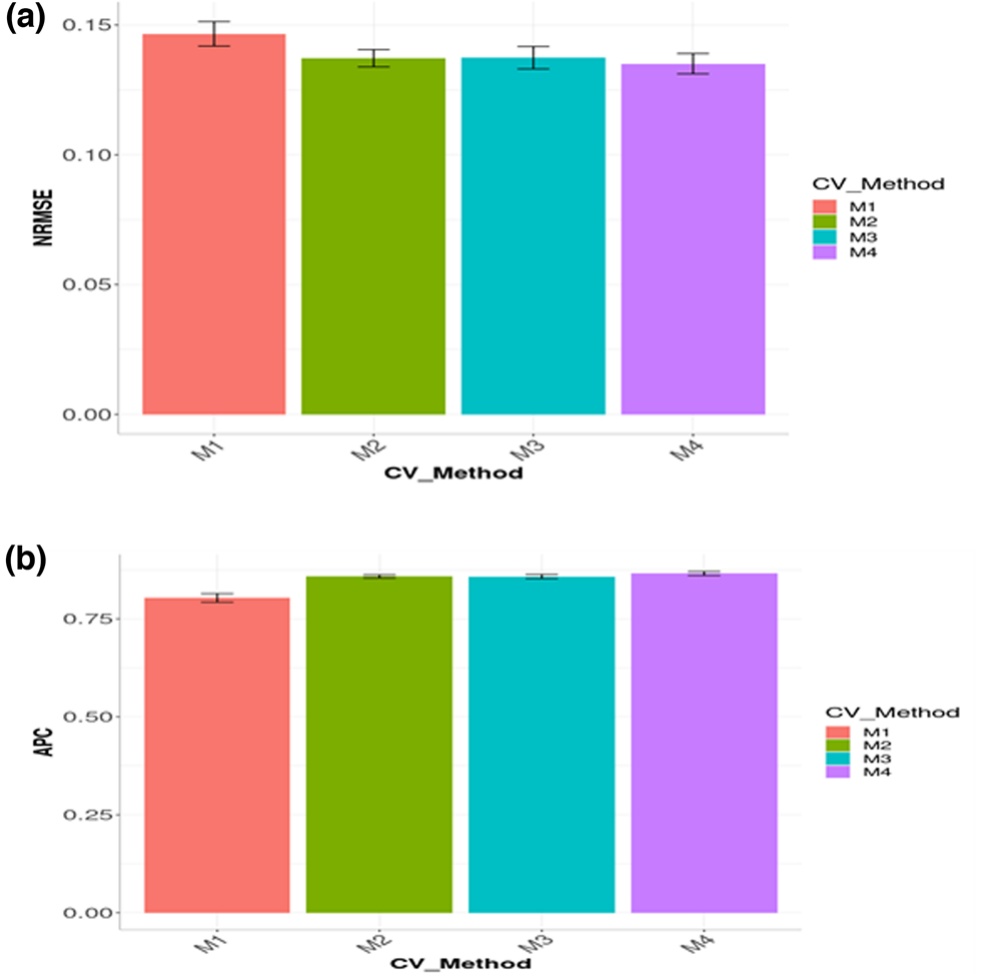
Prediction performance in terms of normalized root mean squared error (NRMSE) (A) and average Pearson's correlation (APC) (B) for data set 7 (Japonica). M1 denotes the allocation of lines in all locations, M2 denotes the allocation with some shared lines in locations, M3 denotes the random allocation of lines to locations, and M4 denotes the allocation of lines to locations using the balanced incomplete block design (BIBD) method. Ten partitions were implemented with 50% of the data for training and 50% for testing

In this data set, we can observe that M4 is superior, in terms of NRMSE, to M1, M2, and M3 by 7.84%, 1.67%, and 1.53%, respectively (Figure 8A). The difference can be seen in Figure 8, and we can observe that M2 and M3 give similar results. In terms of APC (Figure 8B), while M2 and M3 provide similar results, the highest APC is also observed by M4 and the worst by M1, as expected. Details are in Table A1.

## 4 DISCUSSION

A successful implementation of GS strongly depends on its ability to guarantee high prediction accuracy of the genetic merit of candidate genotypes. However, the quality of prediction accuracy strongly depends on the number of replications (or environments) evaluated in plant breeding programs. As such, sparse testing methods offer the opportunity to increase the selection intensity in early yield breeding programs even with the same number of testing environments. For this reason, it is important to evaluate the existing sparse testing strategies to use the more optimal one for the selection of candidate genotypes with high accuracy.

Therefore, we performed a benchmarking study to compare the following four sparse testing methods: M1 where we allocated some lines in all locations, M2 where we allocated a fraction of lines with some shared lines in locations, M3 where the allocation of some lines to location was random, and M4 were the allocation of a subset of lines to locations was performed using a balanced incomplete block experimental design. See Figure 1 for details of these four methods.

We found that the allocation of lines to locations (or environments) using the BIBD method (M4) is superior to the allocation of lines in all locations (M1), allocation with some shared lines in locations (M2), and random allocation of lines to locations (M3) in all the evaluated data sets. With the biggest data sets, the superiority of methods—M4 and M3—over the conventional method (M1) is clearer. For example, in terms of NRMSE, in data set 5 (EYT_4), which is the biggest data set, method M4 outperformed M1 by 15.94%, while for data set 7 (Japonica), the smallest data set, M4 outperformed M1 only by 7.84%. In terms of APC, the following pattern is also observed: M4 gives the greatest APC in all data sets while M1 gives the lowest APC. In general, we observed that the methods M3 and M4 are not affected largely by the size of the data set.

The original method (M4) was initially proposed under a univariate (single‐trait) setting (Montesinos‐López, Montesinos‐Lopez, Acosta, et al., 2022), that is, for univariate genomic prediction and in this publication, it was only compared with method M3. These authors reported that the M4 was superior in terms of prediction accuracy to M3. However, with the goal to study if this proposed M4 can be extrapolated to the multivariate (multi‐trait) context, that is, when the goal is to predict more than one trait, which is very important in genomic prediction since it can help to increase the prediction accuracy taking advantage of the degree of correlation between traits, we compared not only M3 and M4, but also M1 and M2. This study shows that the findings of Montesinos‐López, Montesinos‐Lopez, Acosta, et al. (2022) for single‐trait are also valid under a multi‐trait genomic prediction framework, but now, we did not find very large differences between M3 and M4. However, since these methods (M3 and M4) were also compared to methods M1 and M2, we found that the worst method was M1, followed by M2, while the best method was M4, followed by M3.

### 4.1 Optimizing sparse testing by random allocation of lines in environments

For genomic‐assisted breeding, it is of great importance to optimize markers and field evaluation to increase genetic gains. Breeders define the cost of genotyping in terms of field plot units and then set different strategies for defining the number of lines to genotype and evaluate in the field using a field sparse allocation scheme. Although several strategies could be studied, the main goal of sparse testing scheme is increasing the intensity of selections by increasing the number of testing lines. This strategy requires the investigation of how many candidate lines could be repeatedly tested in environments and how many could be planted in only one or a few environments.

Adequate testing strategies are fundamental to accurately recycle the parents that sparse testing will be seen to obtain a higher selection intensity and larger environmental sampling, as more lines can be tested in more environments. Jarquin et al. (2020) in maize and Crespo‐Herrera et al. (2021) in wheat multi‐environments trials assessed the effects for the genomic predictive ability of allocation designs in which a certain number of different maize or wheat lines are distributed in different environments and another set of lines is repeatedly observed in all environments.

Results of the maize and wheat multi‐environment trials for genomic prediction accuracy indicate that important savings could be achieved by testing a small number of lines in all environments (∼30–50) and allocating the rest of the lines in an overlapping design in different environments. Results showed that those statistical models that included the GE interaction component capture a larger genetic variability than the main genomic effects model and increase the final prediction accuracy by leveraging the interaction via other genetically related cultivars tested in those environments.

Sometimes the size of the testing populations (for all allocation designs) decrease the genomic prediction accuracy, which is recovered when more lines are tested in all environments. Interestingly, the model that includes GE interaction maintains the prediction accuracy throughout both extreme situations of not having repeated lines in all environments or having all repeated lines in all environments.

### 4.2 Optimizing sparse testing balanced incomplete block design—Single trait

Atanda et al. (2021) concluded that for implementing sparse testing GP for multi‐environment yield trials, it is important to consider the proportion of lines that overlap across environments in the early yield testing stages of population improvement. The authors suggested including a substantial number of common lines across environments to reach an accurate estimation of genetic correlation between environments and improve the genomic prediction accuracy of the GE interaction. Jarquin et al. (2020) reported improved predictive ability by increasing the number of lines that overlapped across three environments. The previous studies consider a random allocation of tested lines in environments by guaranteeing a proportion of non‐overlapping/overlapping lines randomly allocated in environments.

Recently, Montesinos‐López et al. (2020) proposed using BIBDs, principally, for the allocation of lines to environments. The authors compared the random allocation of lines to environments with the proposed single trait BIBD allocation and found that allocation under the principle of BIBD outperformed random allocation by between 1.4% and 26.5% across environments, traits, and data sets in terms of mean square error. This was the first evidence that using BIBD for the allocation of lines to environments can help improve genomic predictive performance compared to random allocation.

### 4.3 Optimizing sparse testing by a balanced incomplete block design—Multi‐trait

As mentioned above, sparse methods are helpful for increasing the selection intensity (e.g., testing more genotypes) (with the same level of prediction accuracy) at the population improvement stage one or even at preliminary yield trials, where testcross hybrids are evaluated in —three to five environments, and several experimental hybrids are obtained by crossing double haploid lines (or lines developed using the pedigree scheme) to a tester from a complementary heterotic group (Atanda et al., 2021).

In this case, the sparse testing methods enhance the number of hybrids to be evaluated in the same number of locations without increasing the breeding costs. For this reason, given that in most breeding programs there are many populations with phenotypic and genomic records in different environments, the sparse testing method presented in this study (multi‐trait BIBD) could be an attractive alternative for creating a robust historical data set that can borrow information across environments, and thus resulting in improved prediction accuracy when compared to the test‐half‐predict‐half strategy (Atanda et al., 2020, 2021; Burgueño et al., 2012; Santantonio et al., 2020).

In product profile stage (stage three, advanced yield trials), breeders can maintain the selection intensity and increase the prediction accuracy of the GS methodology without a significant increase in the breeding cost, as such breeders can increase the number of replications of the hybrids at the same locations or increasing the number of testing environments.

One possible explanation why the gain in prediction accuracy using the multi trait BIBD sparse testing could be equal or slightly lower compared with the single trait BIBD sparse testing is because the building process of BIBD sparse multi trait is under a univariate framework and thus not optimal for the BIBD multi trait. Further research is required for developing an optimization method for the multi trait BIBD sparse testing.

## 5 CONCLUSIONS

Due to the need to improve the practical implementation of the GS methodology, sparse testing methods have been proposed to reduce the cost of phenotyping in GS without a significant reduction in prediction accuracy. Therefore, we performed a benchmarking study to compare four existing methods. The comparisons were made using seven real data sets where we found similar results. Furthermore, we found that the best prediction performance was obtained under the balanced incomplete block design (M4), followed by methods M3 and M2. However, our comparison was done under a multi‐trait framework for which the sparse testing methods were not originally proposed. Nevertheless, we found that even in the multi‐trait context, these methods work, even though they maybe not be as efficient as a single trait framework. Our findings provide empirical evidence that the use of sparse testing in the context of multi‐trait prediction is efficient for improving the efficiency of the GS methodology. However, not all sparse testing methods are equally efficient since the best and second‐best methods were M4 (where we allocated lines to locations under a balanced incomplete block design) and M3 (where we allocated some lines to locations randomly), and the worst was method M1 (where some lines are allocated to all locations).

## AUTHOR CONTRIBUTIONS

**Osval A. Montesinos‐López**: Formal analysis; Investigation; Methodology. **Brandon A. Mosqueda‐González**: Investigation; Methodology. **Josafat Salinas‐Ruiz**: Investigation; Methodology. **Abelardo Montesinos‐López**: Formal analysis; Funding acquisition; Investigation; Methodology. **José Crossa**: Conceptualization; Investigation; Methodology.

## CONFLICT OF INTEREST

The authors declare no conflict of interest.

## Data Availability

The phenotypic and genomic data used for the seven data sets employed in this study can be downloaded from https://hdl.handle.net/11529/10548787.

## 1

**TABLE A1:** Prediction performance in terms of normalized mean square error (NRMSE) and average Pearson´s correlation (APC) for the four sparse methods for allocating lines to locations. M1 denotes the allocation of lines in all locations, M2 denotes the allocation with some shared lines in locations, M3 denotes the random allocation of lines to locations, and M4 denotes the allocation of lines to locations using the balanced incomplete block design (BIBD) method. NRMSE_SE denotes the standard error of the NRMSE estimates and APC_SE denotes the standard error of the APC estimates. Ten partitions were implemented with 50% of the data for training and 50% for testing

Dataset	Method	NRMSE	NRMSE_SE	APC	APC_SE
Data set 1 (disease)	M1	0.6134	0.0037	0.1509	0.0084
	M2	0.5431	0.0013	0.4139	0.0029
	M3	0.5296	0.0013	0.4476	0.0040
	M4	0.5272	0.0014	0.4545	0.0028
Data set 2 (EYT_1)	M1	0.0537	0.0001	0.8587	0.0008
	M2	0.0486	0.0001	0.9178	0.0006
	M3	0.0467	0.0001	0.9296	0.0006
	M4	0.0459	0.0001	0.9363	0.0004
Data set 3 (EYT_2)	M1	0.0668	0.0002	0.9238	0.0004
	M2	0.0588	0.0002	0.9459	0.0003
	M3	0.0570	0.0002	0.9497	0.0004
	M4	0.0558	0.0001	0.9523	0.0002
Data set 4 (EYT_3)	M1	0.0559	0.0002	0.6994	0.0026
	M2	0.0472	0.0001	0.8156	0.0014
	M3	0.0460	0.0002	0.8322	0.0016
	M4	0.0448	0.0001	0.8444	0.0009
Data set 5 (EYT_4)	M1	0.0665	0.0003	0.7478	0.0032
	M2	0.0559	0.0002	0.8598	0.0004
	M3	0.0554	0.0002	0.8683	0.0006
	M4	0.0546	0.0002	0.8750	0.0006
Data set 6 (groundnut)	M1	0.3788	0.0025	0.5199	0.0067
	M2	0.3742	0.0022	0.5351	0.0072
	M3	0.3681	0.0019	0.5369	0.0039
	M4	0.3660	0.0023	0.5628	0.0065
Data set 7 (japonica)	M1	0.1466	0.0024	0.8035	0.0056
	M2	0.1372	0.0017	0.8583	0.0021
	M3	0.1374	0.0022	0.8580	0.0027
	M4	0.1351	0.0020	0.8658	0.0026

## References

[tpg220305-bib-0001] Atanda, S. A. , Olsen, M. , Burgueño, J. , Crossa, J. , Dzidzienyo, D. , Beyene, Y. , Gowda, M. , Dreher, K. , Zhang, X. , Prasanna, B. M. , Tongoona, P. , Danquah, E. Y. , Olaoye, G. , & Robbins, K. R. (2020). Maximizing efficiency of genomic selection in CIMMYT's tropical maize breeding program. Theoretical and Applied Genetics Theoretische, 134, 279–294. 10.1007/s00122-020-03696-9 PMC7813723 33037897

[tpg220305-bib-0002] Atanda, S. A. , Olsen, M. , Crossa, J. , Burgueño, J. , Rincent, R. , Dzidzienyo, D. , Beyene, Y. , Gowda, M. , Dreher, K. , Boddupalli, P. M. , Tongoona, P. , Danquah, E. Y. , Olaoye, G. , & Robbins, K. R. (2021). Scalable sparse testing genomic selection strategy for early yield testing stage. Frontiers in Plant Science, 12, 658978 10.3389/fpls.2021.658978 34239521 PMC8259603

[tpg220305-bib-0003] Bradbury, P. J. , Zhang, Z. , Kroon, D. E. , Casstevens, T. M. , Ramdoss, Y. , & Buckler, E. S. (2007). TASSEL: Software for association mapping of complex traits in diverse samples. Bioinformatics, 23(19), 2633–2635. 10.1093/bioinformatics/btm308 17586829

[tpg220305-bib-0004] Burgueño, J. , de Los Campos, G. , Weigel, K. , & Crossa, J. (2012). Genomic prediction of breeding values when modeling genotype × environment interaction using pedigree and dense molecular markers. Crop Science, 52(2), 707–719. 10.2135/cropsci2011.06.0299

[tpg220305-bib-0005] Crespo‐Herrera, L. , Howard, R. , Piepho, H.‐P. , Pérez‐Rodríguez, P. , Montesinos‐Lopez, O. , Burgueño, J. , Singh, R. , Mondal, S. , Jarquín, D. , & Crossa, J. (2021). Genome‐enabled prediction for sparse testing in multi‐environmental wheat trials. Plant Genome, 14, e20151. 10.1002/tpg2.20151 34510790 PMC12806883

[tpg220305-bib-0006] Crossa, J. , Pérez‐Rodríguez, P. , Cuevas, J. , Montesinos‐López, O. , Jarquín, D. , De Los Campos, G. , Burgueño, J. , González‐Camacho, J. M. , Pérez‐Elizalde, S. , Beyene, Y. , Dreisigacker, S. , Singh, R. , Zhang, X. , Gowda, M. , Roorkiwal, M. , Rutkoski, J. , & Varshney, R. K. (2017). Genomic selection in plant breeding: Methods, models, and perspectives. Trends in Plant Science, 22(11), 961–975. 10.1016/j.tplants.2017.08.011 28965742

[tpg220305-bib-0007] Cuevas, J. , Crossa, J. , Montesinos‐López, O. A. , Burgueño, J. , Pérez‐Rodríguez, P. , & de Los Campos, G. (2017). Bayesian genomic prediction with genotype × environment interaction kernel models. G3: Genes|Genomes|Genetics, 7, 41–53. 10.1534/g3.116.035584 27793970 PMC5217122

[tpg220305-bib-0008] Elshire, R. J. , Glaubitz, J. C. , Sun, Q. , Poland, J. A. , Kawamoto, K. , Buckler, E. S. , & Mitchell, S. E. (2011). A robust, simple genotyping‐by‐sequencing (GBS) approach for high diversity species. PLoS One, 6(5), e19379. 10.1371/journal.pone.0019379 21573248 PMC3087801

[tpg220305-bib-0009] Huang, M. , Balimponya, E. G. , Mgonja, E. M. , Mchale, L. K. , Luzi‐Kihupi, A. , Wang, G.‐L. , & Sneller, C. H. (2019). Use of genomic selection in breeding rice (*Oryza sativa* L.) for resistance to rice blast (*Magnaporthe oryzae*). Molecular Breeding, 39, 114. 10.1007/s11032-019-1023-2

[tpg220305-bib-0010] Jarquin, D. , Howard, R. , Crossa, J. , Beyene, Y. , Gowda, M. , Martini, J. W. R. , Covarrubias Pazaran, G. , Burgueño, J. , Pacheco, A. , Grondona, M. , Wimmer, V. , & Prasanna, B. M. (2020). Genomic prediction enhanced sparse testing for multi‐environment trials. G3: Genes|Genomes|Genetics, 10(8), 2725–2739. 10.1534/g3.120.401349 32527748 PMC7407457

[tpg220305-bib-0011] Juliana, P. , Singh, R. P. , Poland, J. , Mondal, S. , Crossa, J. , Montesinos‐López, O. A. , Dreisigacker, S. , Pérez‐Rodríguez, P. , Huerta‐Espino, J. , Crespo‐Herrera, L. , & Govindan, V. (2018). Prospects and challenges of applied genomic Selection—A new paradigm in breeding for grain yield in bread wheat. The Plant Genome, 11(3), 180017. 10.3835/plantgenome2018.03.0017 PMC7822054 30512048

[tpg220305-bib-0012] Lopez‐Cruz, M. , & de Los Campos, G. (2021). Optimal breeding‐value prediction using a sparse selection index. Genetics, 218, 1–10. 10.1093/genetics/iyab030 PMC8128408 33748861

[tpg220305-bib-0013] Lopez‐Cruz, M. , Olson, E. , Rovere, G. , Crossa, J. , Dreisigacker, S. , Mondal, S. , Singh, R. , & Campos, G. d. L. (2020). Regularized selection indices for breeding value prediction using hyper‐spectral image data. Scientific Reports, 10, 1–12. 10.1038/s41598-020-65011-2 32424224 PMC7235263

[tpg220305-bib-0014] Meuwissen, T. H. E. , Hayes, B. J. , & Goddard, M. E. (2001). Prediction of total genetic value using genome‐wide dense marker maps. Genetics, 157, 1819–1829. 10.1093/genetics/157.4.1819 11290733 PMC1461589

[tpg220305-bib-0015] Money, D. , Gardner, K. , Migicovsky, Z. , Schwaninger, H. , Zhong, G.‐Y. , & Myles, S. (2015). LinkImpute: Fast and accurate genotype imputation for nonmodel organisms. G3: Genes|Genomes|Genetics, 5(11), 2383–90. 10.1534/g3.115.021667 26377960 PMC4632058

[tpg220305-bib-0016] Montesinos‐Lopez, O. A. , Montesinos‐Lopez, A. , Acosta, R. , Varshney, R. K. , Bentley, A. , & Crossa, J. (2022). Using an incomplete block design to allocate lines to environments improves sparse genome‐based prediction in plant breeding. Plant Genome, 15, e20194. 10.1002/tpg2.20194 35170851 PMC12807389

[tpg220305-bib-0017] Montesinos‐López, O. A. , Montesinos‐López, A. , & Crossa, J. (2022). Multivariate statistical machine learning methods for genomic prediction. Springer International Publishing. 36103587

[tpg220305-bib-0018] Montesinos‐López, O. A. , Montesinos‐López, J. C. , Singh, P. , Lozano‐Ramirez, N. , Barrón‐López, A. , Montesinos‐López, A. , & Crossa, J. (2020). A multivariate Poisson deep learning model for genomic prediction of count. G3: Genes|Genomes|Genetics, 10(11), 4177–4190. 10.1534/g3.120.401631 32934019 PMC7642922

[tpg220305-bib-0019] Monteverde, E. , Gutierrez, L. , Blanco, P. , Pérez de Vida, F. , Rosas, J. E. , Bonnecarrère, V. , Quero, G. , & Mccouch, S. (2019). Integrating molecular markers and environmental covariates to interpret genotype by environment interaction in rice (*Oryza sativa* L.) grown in subtropical areas. G3: Genes|Genomes|Genetics, 9(5), 1519–31. 10.1534/g3.119.400064 30877079 PMC6505146

[tpg220305-bib-0020] Pandey, M. K. , Chaudhari, S. , Jarquin, D. , Janila, P. , Crossa, J. , Patil, S. C. , Sundravadana, S. , Khare, D. , Bhat, R. S. , Radhakrishnan, T. , Hickey, J. M. , & Varshney, R. K. (2020). Genome‐based trait prediction in multi‐0.167 emenvironment breeding trials in groundnut. Theoretical and Applied Genetics, 133(11), 3101–17. 10.1007/s00122-020-03658-1 32809035 PMC7547976

[tpg220305-bib-0021] Pérez, P. , & De Los Campos, G. (2014). Genome‐wide regression and prediction with the BGLR statistical package. Genetics, 198(2), 483–95. 10.1534/genetics.114.164442 25009151 PMC4196607

[tpg220305-bib-0022] R Core Team. (2022). R: A language and environment for statistical computing. R Foundation for Statistical Computing.

[tpg220305-bib-0023] Rio, S. , Charcosset, A. , Mary‐Huard, T. , Moreau, L. , & Rincent, R. (2022). Building a calibration set for genomic prediction, characteristics to be considered, and optimization approaches. In N. Ahmadi & J. Bartholomé (Eds.), Genomic prediction of complex traits. Methodsx in molecular biology (pp. 77–112), Humana. 10.1007/978-1-0716-2205-6_3 35451773

[tpg220305-bib-0024] Roorkiwal, M. , Rathore, A. , Das, R. R. , Singh, M. K. , Jain, A. , Srinivasan, S. , Gaur, P. M. , Chellapilla, B. , Tripathi, S. , Li, Y. , Hickey, J. M. , Lorenz, A. , Sutton, T. , Crossa, J. , Jannink, J.‐L. , & Varshney, R. K. (2016). Genome‐enabled prediction models for yield related traits in chickpea. Frontiers in Plant Science, 7, 1666. 10.3389/fpls.2016.01666 27920780 PMC5118446

[tpg220305-bib-0025] Santantonio, N. , Atanda, S. A. , Beyene, Y. , Varshney, R. K. , Olsen, M. , Jones, E. , Roorkiwal, M. , Gowda, M. , Bharadwaj, C. , Gaur, P. M. , Zhang, X. , Dreher, K. , Ayala‐Hernández, C. , Crossa, J. , Pérez‐Rodríguez, P. , Rathore, A. , Gao, S. Y. , Mccouch, S. , & Robbins, K. R. (2020). Strategies for effective use of genomic information in crop breeding programs serving Africa and South Asia. Frontiers in Plant Science, 11, 353. 10.3389/fpls.2020.00353 32292411 PMC7119190

[tpg220305-bib-0026] Smith, A. B. , Butler, D. G. , Cavanagh, C. R. , & Cullis, B. R. (2015). Multiphase variety trials using both composite and individual replicate samples: A model‐based design approach. Journal of Agricultural Science, 153, 1017–1029. 10.1017/S0021859614000707

[tpg220305-bib-0027] Smith, A. B. , Ganesalingam, A. , Kuchel, H. , & Cullis, B. R. (2015). Factor analytic mixed models for the provision of grower information from national crop variety testing programs. Tag. Theoretical and Applied Genetics Theoretische Und Angewandte Genetik, 128, 55–72. 10.1007/s00122-014-2412-x 25326722 PMC4282718

[tpg220305-bib-0028] Vanraden, P. M. (2008). Efficient methods to compute genomic predictions. Journal of Dairy Science, 91(11), 4414–23. 10.3168/jds.2007-0980 18946147

[tpg220305-bib-0029] Wolfe, M. D. , del Carpio, D. P. , Alabi, O. , Ezenwaka, L. C. , Ikeogu, U. N. , Kayondo, I. S. , Lozano, R. , Okeke, U. G. , Ozimati, A. A. , Williams, E. , Egesi, C. , Kawuki, R. S. , Kulakow, P. , Rabbi, I. Y. , & Jannink, J.‐L. (2017). Prospects for genomic selection in cassava breeding. Plant Genome, 10(3). 10.3835/plantgenome2017.03.0015 PMC7822052 29293806

